# The enriched Y-bearing sperm combined with delayed fixed-time artificial insemination for obtaining male Simmental crossbred offspring

**DOI:** 10.14202/vetworld.2022.102-109

**Published:** 2022-01-22

**Authors:** Dewa Ketut Meles, Imam Mustofa, Mas’ud Hariadi, Wurlina Wurlina, Suherni Susilowati, Anny Amaliya, Suparto Suparto, Rimayanti Rimayanti

**Affiliations:** 1 Division of Basic Veterinary Medicine, Faculty of Veterinary Medicine, Universitas Airlangga, Surabaya, Kampus C Mulyorejo, Surabaya 601155, East Java, Indonesia; 2 Division of Veterinary Reproduction, Faculty of Veterinary Medicine, Universitas Airlangga, Surabaya, Kampus C Mulyorejo, Surabaya 601155, East Java, Indonesia; 3 The Singosari National Artificial Insemination Center, Ngujung, Toyomarto, Singosari, Malang District 65153, East Java, Indonesia; 4 Gunungrejo Makmur Livestock Cooperative, Kedung Pring, Lamongan District 62272, East Jawa, Indonesia.

**Keywords:** agricultural innovation, farm productivity, motility, pregnancy rate, sperm morphologic abnormality, viability

## Abstract

**Background and Aim:**

The production of male calf beef cattle is an agricultural innovation needed to increase the farm’s productivity as a provider of meat sources. This study aimed to determine the sex ratio of the offspring of cows inseminated with Y-bearing sperm enriched by Percoll density gradient centrifugation and swim-up, combined with delayed fixed-time artificial insemination (FTAI).

**Materials and Methods:**

Ejaculates of Simmental bulls were divided into four equal portions and grouped as T0 (control, non-sexed semen), T1 and T2 were sexed semen using Percoll density gradient centrifugation three and five levels, respectively, and T3 was sexed semen using swim-up. After the sex was sorted, the semen was diluted in a tris-egg yolk extender, packaged in French mini-straws containing 50 million live sperm cells, and frozen. Pre-sexed, post-sexed, and post-thawed spermatozoa were evaluated based on progressive motility, viability, intact plasma membrane, and abnormality. The post-thawed semen of T0 was artificially inseminated to recipient cows at 12 h after onset of estrus (not delayed FTAI). Meanwhile, the delayed FTAI was conducted 18-20 h after onset of estrus using the T0, the best of T1 and T2, and the T3 post-thawed semen.

**Results:**

The Percoll density gradient centrifugation reduced motility, viability, and intact plasma membrane but increased sperm abnormalities. Meanwhile, the swim-up process increased motility, viability, and intact plasma membrane of sperm cells but decreased sperm abnormalities. Post-thawed semen decreased motility, viability, and intact plasma membrane of sperm cells but increased sperm abnormalities. The sex ratio of the Simmental crossbred offspring was 96.08% and 100% in T1 and T3, respectively, compared to 48.25% and 67.39% in T0 not delayed and delayed FTAI, respectively.

**Conclusion:**

The Percoll density gradient centrifugation and swim-up methods are prospective for obtaining male offspring.

## Introduction

Sex manipulation technologies allow predetermination of the offspring’s gender to harvest male calf offspring for increased productivity of farm as the provider of meat source. Sperm separating technologies in sex manipulation can be conducted through Percoll density gradient centrifugation or the swim-up method [1]. The Percoll density gradient centrifugation is based on differences in X- and Y-sperm DNA content, allowing for cell separation. After centrifugation, X-bearing sperm cells settle at the lower layer, while the Y-bearing sperm cells remain at the upper level (supernatant) [2]. Sexing sperm cells using the swim-up method are based on differences in motility speed of swimming out of the pellets to the surface of the media. The Y-bearing sperm cells swim faster than X-bearing sperm cells [3]. The highly motile sperm cells move to the upper level of the medium faster; therefore, the swim-up technique selects the most active and motile sperm cells [4]. Swim-up was proven effective for separating X- and Y-bearing Nili Ravi buffalo bull sperm cells where the expression of Y chromosome-bearing fraction was 4-fold higher in supernatant than control [2].

Natural mating or artificial insemination (AI) using non-sexed semen resulted in a 50%:50% sex ratio. Ovulation occurs approximately 24-32 h after the onset of standing estrus. The fixed-time AI (FTAI) should be conducted 12 h after the onset of estrus in bovine for most sperm cells to reach the ovum at the appropriate time; this process was traditionally called the a.m.-p.m. guideline. When the estrus of cows is observed during a.m. hours, AI should be conducted at the p.m. hours, and vice versa [5]. Since the Y-bearing sperm cells swim faster than the X-bearing sperm cells [3], delayed insemination aims to approach the ovulation time expected to give rise to an XY zygote and then a male calf. Hypothetically, obtaining male calf offspring can be conducted by delaying the FTAI; thereby, the Y-bearing sperm has a higher probability of fertilizing the ovum. There has been no report of using the combination of sexed semen and delayed FTAI to harvest the male Simmental crossbred offspring.

Therefore, this study aimed to determine the sex ratio of the offspring on cows inseminated using enriched Y-bearing sperm using Percoll density gradient centrifugation and swim-up, combined with delayed FTAI.

## Materials and Methods

The experimental procedure was approved by the Animal Care and Use Committee, Airlangga University, Surabaya, Indonesia, No. 267/HRECC.FODM/VII/2019.

### Study period and location

This study was conducted from April 2020 to January 2021. The processing for sexing and freezing of spermatozoa was conducted in the Singosari National Artificial Insemination Center (SNAIC), Malang District, East Java, Indonesia. The SNAIC is located at latitude 7°83’76” S, longitude 112°64’57” E, and altitude 800-1.200 m above sea level (ASL). The climate of Malang District is a climate with the relative humidity is 84%, and temperature is 18-31°C, yearly rainfall is about ± 1.596 mm, and ± 84.85 rainy days per year [6]. The application of post-thawed semen for AI to recipient cows was conducted at Kedungpring, Lamongan District, East Jawa, Indonesia, at latitude S 6°51’54”, longitude E 122°4’44”, and altitude + 23 m ASL. Lamongan District is a tropical climate with a humidity of 64%, average temperature of 27-32°C, yearly rainfall of about ± 1.403 mm, and ± 71.16 rainy days per year [7].

### Experimental design

This study was conducted using a completely randomized design. Each ejaculate was equally divided randomly into four portions for T0 (control, non-sexed semen), T1 and T2 were sexed semen using Percoll density gradient centrifugation three and five levels, respectively, and T3 was sexed semen using swim-up. The recipient cows were 175 head of estrous synchronized local (Peranakan Ongole) cows. They were randomly divided into four groups inseminated with post-thawed semen of T0 at 12 h (not delayed FTAI) and 18-20 h (delayed FTAI) after the onset of estrus, and the best of T1 and T2, as well as T3, was artificially inseminated in local cows at 18-20 h after onset of estrus.

### Animals

Four Simmental bulls and 175 Peranakan Ongole cows were used in this study recipient. The Simmental bulls were aged 3-5 years and weighing 900-950 kg, categorized as superior genetic quality, routinely collected for frozen semen production of the SNAIC. The recipients selected were non-pregnant healthy cows with regular estrus cycles based on farmer record, body condition score of 3-4 (on a scale of 1-5), aged 4-6 years, weighing 550-600 kg, and parity of two to four was used as recipients. The recipient cows were reared at Kedungpring, Lamongan District, East Jawa, Indonesia.

### Extender

Tris-egg yolk extender was prepared freshly by mixing 1.363 g Tris-aminomethane (Merck, CAS Number: 108382, Darmstadt, Germany), 0.762 g citric acid (CAS Number: 77-92-9 100241-Merck Millipore), 1.5 g lactose monohydrate (Merck CAS Number: 1.07660), and 2.7 g D-(+)-raffinose pentahydrate (Merck, CAS Number: 512-69-6), 0.5 g D(-)-fructose (Merck, CAS Number: 57-48-7 104007) was dissolved in 80 mL double-distilled water and homogenized with a magnetic stirrer for 10-15 min [8]. The extender osmolarity was 295 mOsm/L (WESCOR model 5500, INC, USA). The solution was heated to 100°C for a few minutes and cooled to room temperature (26°C), after which it was mixed with 0.1 g penicillin G (Meiji Seika Co., Tokyo, Japan) and 0.1 streptomycin sulfate (Meiji). The mixture was homogenized again for 10-15 min. Egg yolk (20 mL) was dissolved in 80 mL of the mixture, centrifuged 492 *× g* for 15 min, the supernatant was used as an extender while the precipitate was removed [2].

### Semen collection

Ejaculates of Simmental bull were collected using an artificial vagina. The samples were immediately examined for macroscopic (volume, smell, color, viscosity, and pH) and microscopic (mass movement, concentration, sperm motility, viability, morphologic abnormalities, and intact plasma membrane) characteristics [9].

### Semen quality evaluation

The quality of fresh semen, post-sexed, and post-thawed semen was evaluated based on the sperm concentration, viability, motility, IPM, and morphologic abnormality.

### Sperm concentration

The sperm concentration of semen was conducted in a standardized spectrophotometer (Bovine Accucell photometer, IMV, L’Aigle, France). Sperm concentration was determined at 535 nm wavelength using a pre-warmed and calibrated photometer. The photometer was set at zero using normal saline water, and then, 20 mL semen samples were mixed in 2 mL normal saline water [10].

### Sperm viability

A drop of semen, mixed homogenously with eosin-nigrosin on an object-glass, smeared and dried over a flame. The viability of sperm was examined under a light microscope (Olympus BX-53, Olympus Corporation, Tokyo, Japan) at 400×. Live sperm cells had non-stained heads, while dead sperm cells appeared reddish [11].

### Sperm motility

Semen was diluted in physiological saline (0.9% NaCl) (10 mL each), dropped on a warm glass slide, and covered with a coverslip. The progressive movement of sperm cells was assessed under a light microscope at 400 × using a computer-assisted sperm analyzer (CASA, WEI-LI New Century Technical Development, China) [11].

### Plasma membrane intactness

Membrane integrity assessment was conducted using the Hypo Osmotic Swelling test. The hypo-osmotic solution contained 7.35 g sodium citrate 2 _H2_O and 13.52 g fructose dissolved in 1000 mL distilled water (150 mosmol fructose and 150 mosmol sodium citrate). A semen sample of 0.1 mL was added to 1 mL hypo-osmotic solution and incubated for 30 min at 37°C. Cells were observed using a light microscope (Olympus BX-53, Olympus Corporation) at 400×. The curled tails indicated intact plasma membranes, while sperm cells with straight tails indicated damaged membranes [11].

### Sperm morphologic abnormality

A drop of the semen was smeared on a glass slide, allowing it to air dry after which it was stained with eosin nigrosin. The individual sperm was examined using a light microscope (Olympus BX-53, Olympus Corporation) at 400×. Head, neck, and tail morphological defects were evaluated on 100 sperm cells [12].

### Treatment of semen

Each ejaculate was split for semen quality evaluation (1 mL) and treatment groups (the remaining ejaculate volume).

### Non-sexed semen

Three portions of Earle’s balanced salt solution (EBSS, Sigma-Aldrich, Darmstadt, Germany) medium supplemented with 0.1% bovine serum albumin (BSA, Sigma-Aldrich) [4] were prepared for each one portion of fresh semen. Fresh semen 1 mL was pipetted and gently released at the bottom of the 15 mL Falcon tube containing 3 mL EBSS without disturbing the semen-medium interface. Then, the top 3 mL EBSS was immediately discarded [13].

### Discontinuous Percoll density gradient centrifugation preparation

The gradient medium was TCM 199 (Sigma-Aldrich) plus 0.35 g/liter bicarbonate buffer (Sigma-Aldrich). The discontinuous Percoll (Pharmacia, NJ, USA) density gradient was formed by three (90%, 60%, and 30%) and five Percoll concentrations (90%, 75%, 60%, 45%, and 30%). The solutions of those several densities were sequentially arranged in a 15 mL tube from the highest concentrations in the bottom to the lowest concentrations at the top in 2.0 mL each for three levels and 1.2 mL each for five levels of Percoll density gradient medium [14].

One milliliter of fresh semen was put over the last layer of Percoll of three and five gradient concentrations and then centrifuged at 623 *× g* for ten min at room temperature (26°C). Two-third of the upper layer was removed, and the remaining was resuspended with 3 mL EBSS, centrifuged for 5 min at 492 × *g* for sperm washing, followed by discarding 2 mL of the supernatant. The remaining 1 mL pellets were resuspended, washed, and resuspended again with 3 mL EBSS and allowed to stand for 10 min. Finally, 1 mL of the top layer of the supernatant was collected to assess sperm cell quality and for further processing [4].

### Swim-up

A 3 mL EBSS was carefully put into the centrifugation tube wall containing 1 mL fresh semen for swim-up. The tube was tilted to a 45° angle and incubated for 1 h at 27°C to 28.5°C. The tube was carefully reversed to a standing position, then 1 mL of the top layer (supernatant) was harvested for sperm cell quality assessment and further processing [4].

### Freezing, thawing, and sperm post-thaw evaluation

The semen samples were frozen following the Indonesian National Standard (SNI) with the number SNI: 4869-1:2017 (production and analysis of frozen semen) of the National Standardization Agency of Indonesia [15]. The semen was diluted in a tris-egg yolk extender, after which it was equilibrated at 5°C in a cold handling cabinet (Minitüb, Germany) for 22 h[16] after which it was packaged in 0.25 mL French straws containing 50 × 10^6^ live spermatozoa and sealed. The straws were exposed to liquid nitrogen vapor (−140°C) for 10 min and stored in liquid nitrogen (−196°C) for a month before they were assessed and used for AI [17].

The straws of each group were randomly selected for the assessment of post-thawed sperm quality in six replicates. Straws were thawed in sterile water at 37°C for 30 s to assess the sperm motility and viability, plasma membrane integrity, and morphological abnormality.

### Estrus synchronization, AI, and pregnancy diagnosis

The determination of the recipient cows number inseminated was based on the agreement between the research team and the breeders. Transrectal palpation was conducted to ensure that the cows were not pregnant and had a corpus luteum on the ovary. Estrus synchronization was conducted by intramuscular injection of 5 mg prostaglandin F2 alpha (Enzaprost-T, France) twice within 11 days [18]. Two days after the injection, estrus signs were observed. The straws of each group were randomly selected for AI. The post-thawed semen of the T0 group was artificially inseminated in 50 estrus cows at 12 h [5] (control group – not delayed and T0 – not delayed FTAI) and in 50 estrus cows at 18-20 h (control group – delayed and T0 – delayed FTAI) after the onset of estrus. The best quality of post-thawed semen among the T1 and T2 group was artificially inseminated in 50 estrus cows. The T3 (swim-up) group was artificially inseminated in estrus cows at 18-20 h after onset of estrus. Re-insemination was conducted a cycle later when the cow failed to be pregnant at the first AI. Pregnancy diagnosis was performed by transrectal palpation on 90 days post-AI [18].

### Statistical analysis

Progressive motility, viability, IPM, and sperm morphological defects data were analyzed using analysis of variance, followed by the Tukey honestly significant difference. Meanwhile, the sex ratio among groups was analyzed using the Chi-square test. The statistical analysis was conducted at a 95% significance level using Statistical Package for the Social Sciences Version 23 (IBM Corp., NY, USA).

## Results

The characteristics of the fresh semen are presented in Table 1. The post-sexed semen with Percoll density gradient centrifugation (T1 and T2) and swim-up (T3) changed most semen quality parameters compared to T0. Spermatozoa concentrations in all sexing sperm groups were lower than the T0 (p<0.05), with T3 being the lowest. Progressive motility on T1 and T2 was lower than those of T0. Meanwhile, sperm progressive motility of T3 was not significantly different (p>0.05) compared to T0. The sperm viability and IPM of T1 and T3 were lower (p<0.05), while T3 was higher (p<0.05) compared to T0. Sperm morphological abnormality of T1 and T2 was higher, while T3 was lower (p<0.05) than T0. Overall, sperm motility, viability, and IPM of the T2 group were the lowest (p<0.05) compared to the other groups. Meanwhile, the concentration of spermatozoa and sperm morphological abnormalities in the T3 group showed the lowest value [Table 2].

**Table-1:** The Simmental bulls fresh semen quality (n=4 ejaculates).

Parameters	Mean±SD
Volume (mL)	6.88±0.23
pH	6.80±0.44
Sperm motility (%)	85.60±2.50
Sperm concentration (10^6^/mL)	1862.2±95.09
Total number of spermatozoa/ejaculate	12331.71±872.11
Sperm viability sperm (%)	90.80±2.68
Sperm morphological defects (%)	7.80±1.30
Plasma membrane integrity (%)	82.80±2.68

**Table-2:** The comparison of Simmental bulls semen quality without and sexing semen (n=6 replicate).

Group	Concentration	Motility	Viability	IPM	Abnormality
T0	1792.40±126.76^a^	84.20±1.92^a^	86.60±4.27^b^	81.80±2.58^b^	7.40±1.14^b^
T1	1507.80.2±86.09^c^	77.80±0.83^b^	80.80±1.64^c^	78.80±0.83^c^	8.80±1.05^a^
T2	1610.60±140.37^b^	75.40±3.84^c^	76.80±2.38^d^	73.60±3.64^d^	9.40±1.51^a^
T3	1265.8±127.01^d^	85.20±3.89^a^	88.20±2.38^a^	83.40±1.51^a^	6.20±1.30^c^

T0=Control group, T1 and T2 were sexed using Percoll medium of three and five gradient levels, T3=Swim-up sexed semen. Different superscripts in the same column show a significant difference at *P<*0.05

Sperm motility, viability, and IPM were lower, but the abnormality of post-thawed [Table 3] was higher than the pre-freezing of post-sexed semen [Table 2]. Based on the sexing treatment group, the sperm motility and viability of three levels (T1) and five levels (T2) of Percoll density gradient centrifugation were lower (p<0.05) than those of the control group (T0) and the swim-up group (T3). The lowest sperm IPM was T2 (p<0.05) compared to the other group. There were no significant differences in sperm IPM among the T0, T1, and T2 groups. The difference in sperm abnormality data among groups was inverse (p<0.05) to the sperms motility and viability data. There were no significant differences (p>0.05) in the number of motile sperm among groups.

**Table-3:** Quality and the number of motile post-thawed sperm cells per straw of the sexed Simmental bull semen (n=6).

Group	Motility	Viability	Plasma membrane intactness	Abnormality	Number of motile sperm (×10^6^)
T0	48.56±1.08^a^	52.19±2.91^a^	44.11±1.78^a^	12.79±1.18^b^	24.28±5.26
T1	44.52±0.79^b^	48.55±1.53^b^	42.48±1.26^a^	14.39±0.21^a^	22.26±3.58
T2	43.36±2.15^b^	46.12±1.77^b^	39.76±2.12^b^	15.19±1.63^a^	21.68±1.62
T3	49.14±2.76^a^	53.41±2.67^a^	45.20±2.45^a^	11.19±1.27^b^	24.57±3.42

T0=Control group, T1 and T2 were sexed using Percoll medium of three and five gradient levels, T3=Swim-up sexed semen, different superscripts in the same column shows a significant difference at *P<*0.05.

Two of the 50 cows in the T0 delayed FTAI group were sold by the owner before insemination, and two of 25 cows of the T3 group failed to get pregnant up to the second insemination. There were no significant differences (p>0.05) in the pregnancy rate among groups. The sex ratio of the Simmental crossbred offspring was 52% in the T0 not delayed FTAI group and 67.39% in the T0 delayed FTAI group (p>0.05). The combination treatment of sexing sperm using Percoll density gradient centrifugation (T1) with delayed FTAI and swim-up (T3) with delayed FTAI resulted in sex ratio offspring 96.08% and 100% (p>0.05), both were higher (p<0.05) than those of T0 delayed and T0 not delayed FTAI [Table 4].

**Table-4:** The pregnancy and sex ratio of the offspring.

Group	n	Pregnancy rate	Calves gender		Sex ratio

			Male	Female	
T0 not delayed FTAI	50	100% (50/50)	24	26	52%^a^
T0 delayed FTAI	50*	96% (46/48)	31	15	67.39%^a^
T1	50	90% (45/50)	49**	2	96.08%^b^
T3	25***	92% (23/25)	23	0	100%^b^

Sex ratio=Is the percentage of male calves per calving.

* Two of 50 cows were sold by the owner before inseminated,

** there was a pair of twins,

*** two of 25 cows failed to pregnant up to the second insemination. Different superscripts in the same column show a significant difference (p<0.05). FTAI=Fixed-time artificial insemination

## Discussion

The average concentration was above 600 million/mL, progressive motility was above 70%, and abnormalities were less than 20%, indicating that the ejaculates were qualified for frozen semen production [15]. Treatment for sort sexing semen causes a decrease in sperm concentration. In the swim-up method, only motile sperm cells move to the upper level of the medium that causes lower sperm concentration [2]. Meanwhile, the Percoll gradient density centrifugation generated reactive oxygen species (ROS), impairing the structural and functional integrity of sperm cells, followed by mitochondrial and DNA damage, and finally, sperm death [4]. The decrease in sperm concentration was there because some populations of sperm were dead and remained in the bottom of the tube.

Some parameter results of this study were not following various previous reports that Percoll gradient treatment increases sperm motility [14], percentage of cells with normal morphology [19], and intactness of membrane [20]. This may be due to the fact that only Y-bearing spermatozoa were harvested in this study. The Percoll density gradient centrifugation differentiates sperm cells based on their density. After centrifugation, the highly motile, sperm morphologically normal, and viable sperm cells was formed a pellet at the bottom of the tube. The centrifugation in the Percoll density gradient centrifugation method caused a low percentage of sperm cell motility due to friction between the Percoll particles and the sperm cells, which damaged the sperm cell membrane. The sperm plasma membranes’ damage causes the metabolic process to be interfered so that the ATP of sperm cells is low [21].

The sperm viability of the Percoll density gradient centrifugation method was lower than those of the non-sexed semen group. The dead sperm cells cannot migrate to the separating medium. Some immotile and morphologically abnormal live sperm cells also filtered through the separating medium, reducing the number of viable sperm cells [21].

The IPM of sperm was reduced in the Percoll density gradient centrifugation group compared to the control group. This occurred due to mechanical friction between sperm cells’ membrane surface and the Percoll particles or tube walls during centrifugation. The friction caused deformation of the extracellular matrix, including changes in the membrane’s lipid composition, which was essential to maintain permeability and fluidity of the sperm cell membrane [22]. The absence of seminal plasma caused the sperm cell plasma membrane to become less stable. Seminal plasma contains sodium, potassium, protein, ascorbic acid, and many nutrients. Ascorbic acid, an antioxidant, keeps the plasma membrane from oxidative phosphorylation [23]. Meanwhile, sodium and potassium ions in seminal plasma play a key role in sperm metabolism through the plasma membrane [24]. Protein in seminal plasma functions as a jacket to protect the plasma membrane, thereby retaining flexibility and preventing sperm cells from irreversible damage [25].

Sperm cell abnormalities in sexed semen using the Percoll density gradient centrifugation were higher than those of the swim-up method due to damaged sperm cells’ membrane during the processes. The alterations in ion concentration damage the morphology of sperm cells. Furthermore, abnormal sperm cells can be caused by exposure to toxic substances, either from dead sperm cells or substances in diluents oxidized due to storage. In addition, high levels of free radicals cause damage to the sperm cell plasma membrane [12].

The swim-up technique selects the most active and motile sperm cells [4]. This method is based on the swimming speed differences between X- and Y-bearing sperm cells. The highly motile sperm cells migrate to the top of the medium. Swim-up could effectively separate X- and Y-bearing sperm cells of the Nili Ravi buffalo bull [2]. The viability of sexed semen using the swim-up method was higher than those of the non-sexed group. Live sperm cells move out of the pellets to the medium’s surface, whereas dead sperm cells remain in the lower layer. The swim-up method separates the living and highly motile sperm cells from dead, immotile, and abnormal sperm cells. Sexing sperm cells using the swim-up method causes a low concentration of sperm cells because only motile sperm cells will be able to move upwards to the media’s surface. Sperm washing using the swim-up method caused a decrease in sperm concentration, increased sperm motility, and decreased abnormal sperm cells post-swim-up compared to sperm cells before swim-up [26]. Several factors contribute to maintaining sperm cell motility; ATP is an energy source for sperm cells. The difference between Y- and X-bearing sperm cells is their DNA content, which is responsible for differences in the expression of genes and proteins for specific sexes [27]. Toll-like receptors 7/8 (*TLR7/8*), encoded by the X-bearing chromosome, suppressed the motility of X-bearing sperm cells without altering their fertilization ability [3]. Unfortunately, the swim-up method increased sperm cell DNA fragmentation to approximately 40-60%, which caused the lower conception [28].

The freezing and thawing process of semen decreased sperm cell motility, viability, and IPM and increased the percentage of abnormal sperm cells. During freezing, semen is exposed to cold shock and atmospheric oxygen, which causes higher ROS production [29]. The sperm cell plasma membrane is first affected by the freezing process. The sperm cell membrane consists of polyunsaturated fatty acids, which are susceptible to lipid peroxidation by ROS. Lipid peroxidation causes membrane integrity loss with increased permeability, reduced sperm viability, and motility. Sperm cells need a low dose of ROS for a physiologic role in tyrosine phosphorylation, sterol oxidation, and cholesterol efflux during capacitation and fertilization. High ROS levels will disrupt the electron transport chain flow, which results in electron leakage in the mitochondria. The plasma membrane’s intactness is essential for sperm cells as it affects the metabolism associated with motility and viability [30]. During thawing, sperm cells undergo extreme alterations in temperature and osmolarity. Osmotic changes caused damage to the lipid membrane structure [31]. The penetration rates, cleavage, and blastocyst formation were significantly improved and positively influenced by the intactness of sperm cell head membranes [19].

The pregnancy of the T0 delayed FTAI cows (96.08%) was higher compared to a previous study (83.33%) for cows inseminated with the Simmental bull non-sexed post-thawed semen [18]. This result was also higher than 40.1-52.8% conception rates for post-insemination using sexed semen [32]. The dosage of sperm cells packaged in a straw for freezing ranged from 20[33] to 100 million [34]. In this study, the semen packaged 50 million sperm cells in a straw, which resulted in approximately twice the final post-thawed sperm cell motility of the 10 million as required. Altogether, the higher pregnancy percentage was due to a higher dose of motile sperm. However, several cows artificially inseminated with post-thawed sexed semen failed to get pregnant. Sexed semen using centrifugation-based methods affected DNA damage mediated to ROS production [4]. As well centrifugation-based methods, the swim-up method increased sperm cell DNA fragmentation which caused the lower conception [28]. The addition of an antioxidant increased the quality and reduced DNA mutation of post-thawed sperm [35]. The lower the sperm DNA fragmentation resulted in a higher pregnancy rate [18].

Usually, the proportion of male and female offspring is approximately 50% each because the population of sperm cells contains the Y chromosome or X chromosome in the same proportion [3]. The sexing semen aims are to shift the ratio of X/Y-bearing sperm cells as desired. The Percoll density gradient centrifugation method settled the X-bearing sperm cells in the lower layer at 60.75%, while the Y-bearing sperm cells were obtained at the upper (supernatant) [2]. The Percoll density gradient centrifugation shifted the ratio of X-and Y-bearing sperm cells to 28%:72%, respectively [36]. The Y-bearing sperm cells swim-up faster than the X-bearing sperm cells [3]. Therefore, the swim-up method is used to harvest the most motile sperm cells in the upper level of the medium [4]. Since the swim-up method is more efficient for separating X and Y sperm cells [2], we combined the enriched Y-bearing sperm cells in the semen with delayed insemination to obtain more male calves.

Obtaining male calf offspring was conducted by giving a greater chance for Y-bearing sperm to fertilize ovum than with X-bearing sperm. This is based on the difference in swimming speed between Y-bearing and X-bearing sperm. Mitochondrial and glycolytic ATP production occurs regardless of TLR 7/8 ligand in the Y-bearing sperm cell. In X-bearing sperm cells, the activity of TLR8 suppressed mitochondrial ATP production, whereas TLR7 affects the phosphorylation of NFkB and GSK3a/b but suppresses hexokinase activity. As a result, ATP production in X-bearing sperm cells was lower due to the *TLR7/8* ligand condition. Based on this mechanism, Y-bearing sperm cells showed a higher motility speed than X-bearing sperm cells [3]. The vagina’s acidity during fertilization may influence the migration of X- and Y-bearing sperm cells, leading to skewness in the sex offspring [37]. The lowest intrauterine pH was at 8 h of estrus and increased 16-24 h later [38]. It was observed that an alkaline pH is more favorable for the survival of Y-bearing sperm cells [39]. Furthermore, the delayed insemination 18-20 h after estrus onset is expected to allow the Y-bearing sperm cells to reach the site of fertilization, which has an alkaline pH. These observations were used as a basis for delayed FTAI to obtain XY zygote.

The estrus expression is essential for conception rates. Estrus detection is accomplished using cow’s standing estrus [5]. Higher pregnancy rates to AI were obtained among cows that expressed estrus before AI [40]. Ovulation occurs approximately 24-32 h after the onset of standing estrus. The conception rates were similar between normal and sex-sorted semen when estrus was expressed before FTAI [41]. This study used a portion of sexed semen presumed to contain more Y-bearing sperm cells combined with delaying FTAI to ensure more male offspring. The pregnancy rate of cows inseminated with sexed semen was not affected by delayed insemination of approximately 12 h [40]. The sperm cells move through the cervix and uterus to infiltrate the oviduct in numbers sufficient for oocyte fertilization in 6-8 h. Ovulation occurs 25-32 h after the onset of standing heat [5]. Thus, within 18-20 h plus 6–8 h of travel, the sperm cells with the fastest motility (Y-bearing sperm cells) swim from the cervix to the fertilization site. This period coincides with ovulation so that a male calf is expected to be obtained. Sex selection has a significant economic impact on herd capacity in beef production. The accuracy of sex selection using AI with sexed semen exceeded 90% [32]. Spermatozoon is a male germ cell that carries the genetic information for determining the sex of the offspring [27].

This study is feasible in field conditions because the implementation of AI of this study was conducted in field conditions. The recipient cows of this study were owned by the farmers who were members of Gunungrejo Makmur Livestock Cooperative at Kedungpring, Lamongan District, East Jawa, Indonesia. Unfortunately, the sex ratio of spermatozoa was not examined before and after the sexing process due to the limited time because this project must be completed in 1 year. Further research is needed to determine sperm sex ratio using polymerase chain reaction as reported by Khamlor *et al*.[42] and its application in a larger recipient population.

## Conclusion

The enriched Y-bearing sperm using Percoll density gradient centrifugation or swim-up methods and the process of freeze-thawing changed sperm motility, viability, integrated plasma membrane, and sperm morphology. However, the quality of semen was still worth using for AI on recipient cows. The Percoll density gradient centrifugation and swim-up method, combined with delayed FTAI, were prospective for harvesting the Y-bearing sperm cells to obtain male offspring.

## Authors’ Contributions

DKM, IM, RR, and WW: Compiled ideas, designed the study, and drafted the manuscript. SS and AA: Processed sexing and evaluated the post-thawed semen fertility. SuS and MH: Prepared and applied AI to the recipients. DKM and IM: Conducted the statistical analysis and drafted the manuscript. RR and MH: Critically read and revised the manuscript for intellectual content. All authors read and approved the final manuscript.

## References

[1] Xie Y, Xu Z, Wu Z, Hong L (2020). Sex manipulation technologies progress in livestock:A review. Front. Vet. Sci.

[2] Asma-Ul-Husna A, Awan M.A, Mehmood A, Sultana T, Shahzad Q, Ansari M.S, Rakha B.A, Saqlan Naqvi S.M, Akhter S (2017). Sperm sexing in Nili-Ravi buffalo through modified swim-up:Validation using SYBR^®^ green real-time PCR. Anim. Reprod. Sci.

[3] Umehara T, Tsujita N, Shimada M (2019). Activation of toll-like receptor 7/8 encoded by the X chromosome alters sperm motility and provides a novel, simple technology for sexing sperm. PLoS Biol.

[4] Meitei H.Y, Uppangala S, Sharan K, Chandraguthi S.G, Radhakrishnan A, Kalthur G, Schlatt S, Adiga S.K (2021). A simple, centrifugation-free, sperm-sorting device eliminates the risks of centrifugation in the swim-up method while maintaining functional competence and DNA integrity of selected spermatozoa. Reprod. Sci.

[5] Stout T.A.E (2018). Female Reproduction in Encyclopedia of Reproduction.

[6] Malang Regency Government (2016). Regional Regulation on Development Plan Regional Medium Term 2016-2021.

[7] Central Bureau of Statistics of Lamongan Regency. Lamongan Regency (2021). Lamongan Regency in Figures. Central Bureau of Statistics of Lamongan Regency, Lamongan Regency.

[8] Rostami B, Ebrahimi D, Sadeghipanah H, Masoumi R, Shahir M.H (2020). Effects of supplementation of tris-egg yolk extender with different sugars and antioxidants on freezability of ram semen. Cryobiology.

[9] Susilowati S, Triana I.N, Wurlina W, Arimbi A, Srianto P, Mustofa I (2019). Addition of L-arginine in skim milk extender maintains goat spermatozoa quality in chilled temperature for five days. Vet. World,.

[10] Rehman S, Shafique L, Yousuf M.R, Liu Q, Ahmed J.Z, Riaz H (2019). Spectrophotometric calibration and comparison of different semen evaluation methods in Nili-Ravi buffalo bulls. Pak. Vet. J.

[11] Susilowati S, Mustofa I, Wurlina W, Triana I.N, Utama S, Rimayanti R (2021). Effect of insulin-like growth factor-1 complex of Simmental bull seminal plasma on post-thawed Kacang buck semen fertility. Vet. World.

[12] Oumaima A, Tesnim A, Zohra H, Amira S, Ines Z, Sana C, Intissar G, Lobna E, Ali J, Meriem M (2018). Investigation on the origin of sperm morphological defects:Oxidative attacks, chromatin immaturity, and DNA fragmentation. Environ. Sci. Pollut Res. Int.

[13] Meena C, Natachandra C, Amiya M, Rao K.A, Srinivas S.R (2006). Sperm preparation in assisted conception:Laboratory techniques. Laboratory Manual in Assisted Reproductive Technology.

[14] Yang Q, Zhang N, Zhao F, Zhao W, Dai S, Liu J, Bukhari I, Xin H, Niu W, Sun Y (2015). Processing of semen by density gradient centrifugation selects sperm with longer telomeres for assisted reproduction techniques. Reprod. Biomed. Online.

[15] INSA (Indonesian National Standard Agency) (2017). Frozen Semen-Part 1:Bovine Bull.

[16] Dwinofanto H, Rimayanti R, Mustofa I, Susilowati S, Hernawati T (2018). The effect of duration of preservation on the quality, MDA level, and DNA damage of post-thawed Bali cattle bull sperm. Iraqi J. Vet. Sci.

[17] Susilowati S, Triana I.N, Sardjito T, Suprayogi T.W, Wurlina W, Mustofa I (2020). Effect of Simmental bull seminal plasma protein in egg yolk-citrate extender on Kacang buck semen fertility. Cryobiology.

[18] Susilowati S, Sardjito T, Mustofa I, Widodo O.S, Kurnijasanti R (2021). Effect of green tea extract in extender of Simmental bull semen on pregnancy rate of recipients. Anim. Biosci.

[19] Oliveira L.Z, Arruda R.P, Celeghini E.C, de Andrade A.F, Perini A.P, Resende M.V, Miguel M.C, Lucio A.C, de Lima V.F.H (2012). Effects of discontinuous percoll gradient centrifugation on the quality of bovine sperm evaluated with computer-assisted semen analysis and fluorescent probes association. Andrologia.

[20] Noguchi M, Yoshioka K, Hikono H, Iwagami G, Suzuki C, Kikuchi K (2015). Centrifugation on PDGC enhances motility, membrane integrity and *in vitro* fertilizing ability of frozen-thawed boar sperm. Zygote.

[21] Assumpção T.I.D, Severo N.C, Zandonaide J. P. B, Macedo G.G (2021). Magnetic-activated cell sorting improves high-quality spermatozoa in bovine semen. J. Anim. Reprod. Biotechnol.

[22] Evans H.C, Dinh T, Hardcastle M.L, Gilmore A.A, Ugur M.R, Hitit M, Jousan F.D, Nicodemus M.C, Memili E (2021). Advancing semen evaluation using lipidomics. Front. Vet. Sci.

[23] Paul R.K, Kumar D, Naqvi S (2017). Antioxidants protect proteins'anchorage to the bilayer by improving plasma membrane integrity of ram sperm during liquid preservation in a soya lecithin-based diluent. Reprod. Domest. Anim.

[24] Bubenickova F, Postlerova P, Simonik O, Sirohi J, Sichtar J (2020). Effect of seminal plasma protein fractions on stallion sperm cryopreservation. Int. J. Mol. Sci.

[25] Pardede B.P, Agil M, Supriatna I (2020). Protamine and other proteins in sperm and seminal plasma as molecular markers of bull fertility. Vet. World.

[26] Fácio C.L, Previato L.F, Machado-Paula L.A, Matheus P.C, Filho A.E (2016). Comparison of two sperm processing techniques for low complexity assisted fertilization:Sperm washing followed by swim-up and discontinuous density gradient centrifugation. JBRA Assist. Reprod.

[27] Rahman M.S, Pang M.G (2020). New Biological insights on X-and Y chromosome-bearing sperm. Front. Cell Dev. Biol.

[28] Muratori M, Tarozzi N, Carpentiero F, Danti S, Perrone F.M, Cambi M, Casini A, Azzari C, Boni L, Maggi M, Borini A, Baldi E (2019). Sperm selection with density gradient centrifugation and swim up:Effect on DNA fragmentation in viable sperm. Sci. Reprod.

[29] Bansal A.K, Bilaspuri G.S (2010). Impacts of oxidative stress and antioxidants on semen functions. Vet. Med. Int.

[30] Takeda K, Uchiyama K, Kinukawa M, Tagami T, Kaneda M, Watanabe S (2015). Evaluation of sperm DNA damage in bulls by TUNEL assay as a parameter of semen quality. J. Reprod. Dev.

[31] Hezavehei M, Sharafi M, Kouchesfahani H.M, Henkel R, Agarwal A, Esmaeili V, Shahverdi A (2018). Sperm cryopreservation:A review on current molecular cryobiology and advanced approaches. RBMO.

[32] Naniwa Y, Sakamoto Y, Toda S, Uchiyama K (2018). Bovine sperm sex-selection technology in Japan. Reprod. Med. Biol.

[33] Karan P, Mohanty T.K, Kumaresan A, Bhakat M, Baithalu R.K, Verma K, Kumar S, Das Gupta M, Saraf K.K, Gahlot S.C (2018). Improvement in sperm functional competence through modified low-dose packaging in French mini straws of bull semen. Andrologia.

[34] Mohanty T.K, Lone S.A, Kumaresan A, Bhakat M, Kumar R, Baithalu R.K, Ranjana Sinha R, Paray A.R, Yadav H.P, Sahu S.G, Mohanty A.K (2018). Sperm dosage and site of insemination in relation to fertility in bovines. Asian Pac. J. Reprod.

[35] Mustofa I, Susilowati S, Wurlina W, Hernawati T, Oktanella Y (2021). Green tea extract increases the quality and reduced DNA mutation of post-thawed Kacang buck sperm. Heliyon.

[36] Kusumawati E.D, Isnaini N, Yekti A.P, Luthfi M, Affandhy L, Pamungkas D, Kuswati K, Ridhowi A, Sudarwati H, Rahadi S, Rahayu S, Susilawati T (2019). The motility and ratio of X and Y sperm filial ongole cattle using different sexed semen methods. Am J. Anim. Vet. Sci.

[37] Raval N.P, Shah T.M, George L.B, Joshi C.G (2019). Effect of the pH in the enrichment of X or Y sex chromosome-bearing sperm in bovine. Vet. World,.

[38] Tsiligianni T, Amiridis G.S, Dovolou E, Menegatos I, Chadio S, Rizos D, Gutierrez-Adan A (2011). Association between physical properties of cervical mucus and ovulation rate in superovulated cows. Can. J. Vet. Res.

[39] Oyeyipo I.P, van der Linde M, du Plessis S.S (2017). Environmental exposure of sperm sex-chromosomes:A gender selection technique. Toxicol. Res.

[40] Chebel R.C, Cunha T (2020). Optimization of timing of insemination of dairy heifers inseminated with sex-sorted semen. J. Dairy Sci.

[41] Crites B.R, Vishwanath R, Arnett A.M, Bridges P.J, Burris W.R, McLeod K.R, Anderson L.H (2018). Conception risk of beef cattle after fixed-time artificial insemination using either SexedUltra™4M sex-sorted semen or conventional semen. Theriogenology.

[42] Khamlor T, Pongpiachan P, Sangsritavong S, Chokesajjawatee N (2014). Determination of sperm sex ratio in bovine semen using multiplex real-time polymerase chain reaction. Asian-Australas J. Anim. Sci.

